# Gender differential item functioning analysis in measuring computational thinking disposition among secondary school students

**DOI:** 10.3389/fpsyt.2022.1022304

**Published:** 2022-11-24

**Authors:** Saralah Sovey, Kamisah Osman, Mohd Effendi Ewan Mohd Matore

**Affiliations:** ^1^Sungai Ramal Secondary School, Bangi, Selangor, Malaysia; ^2^Faculty of Education, Centre of STEM Enculturation, National University of Malaysia, Bangi, Selangor, Malaysia; ^3^Faculty of Education, Research Centre of Education Leadership and Policy, National University of Malaysia, Bangi, Selangor, Malaysia

**Keywords:** computational thinking, disposition, Rasch model, gender differential item functioning, secondary school, student

## Abstract

Computational thinking refers to the cognitive processes underpinning the application of computer science concepts and methodologies to the methodical approach and creation of a solution to a problem. The study aims to determine how students’ cognitive, affective, and conative dispositions in using computational thinking are influenced by a gender. This study used a survey research design with quantitative approach. Five hundred thirty-five secondary school students were sampled using probability sampling with the Computational Thinking Disposition Instrument (CTDI). WINSTEPS version 3.71.0 software was subsequently employed to assess the Gender Differential item functioning (GDIF) including reliability and validity with descriptive statistics were employed to assess students’ disposition toward practicing computational thinking. In addition to providing implications for the theory, the data give verifiable research that the CT disposition profile consists of three constructs. In addition, the demonstrated CTDI has good GDIF features, which may be employed to evaluate the efficacy of the application of CT in the Malaysian curriculum by measuring the level of CT in terms of the disposition profile of students.

## Introduction

Computational thinking (CT) is a vital skill in any field. A number of researchers have proposed that CT serves as a stepping stone to more complex computational endeavors like programming (1). In particular, CT aid elementary school kids in conceptualizing computational reasoning. This is an ability that develops via repeated use (1). The educators viewed technology as a means to broaden their pupils’ horizons and give them more agency in their own learning (2). Recent discussions have centered on the importance of introducing computer science to students in lower grades (3–6). The enthusiasm for CT in the classroom is understandable, but there are still many challenges that must be overcome. As a result, there is a growing expectation that teachers will be able to illustrate computational thinking by applying it to real-world scenarios that use computer technology.

Thus, an item is the fundamental unit of an instrument. The creation of items must be consistent and fair for all participants. DIF refers to a measurement instrument with multiple functions. It is being administered to a group of respondents with diverse demographic backgrounds but comparable abilities. Hambleton and Jones (7) suggest that a DIF-detected item’s functions in various subgroups are dissimilar.

Consequently, the DIF analysis procedure identifies items that do not mirror similar functions when applied to a group of capabilities with parallel capabilities. Osterlind (8) states that item analysis entails observing items critically to reduce measurement error. Consequently, DIF analysis determines item validity (9). DIF endorsement in instrument construction is indicative of an instrument with high reliability. Siti Rahayah Ariffin (10), stated that DIF impacts the dependability of instruments. For composite measures to be unidimensional and the variable to be linear, the item scale values must be consistent across individuals and groups. Three DIF endorsement methods are Mantel-Haenszel (11, 12), Item Response Theory (13), and Rasch Models (14).

We are currently working on the next iteration of CT disposition instruments. Empirical evidence is essential for the creation of new statistical tools. As a result, the gender gap is one of the topics that has gained a lot of attention in academia, especially in the field of computer science education. Since many of the same ideas are used in both CT and computer science, a number of recent studies have looked into the disparity between the sexes in terms of CT proficiency.

From a neurological point of view, boys are a few weeks behind girls and remain behind girls until late adolescence (15). This developmental difference impacts their early school learning experiences and has impact throughout their education. Boys’ fine motor skills develop slower than girls and they may have difficulty with handwriting tasks (16). Their language and fine motor skills fully mature about six years later than girls (17). However, the areas involved in targeting and spatial memory mature some four years earlier in boys than they do in girls (17). Although those differences are significant, it is important to examine how that information relates to developmental gender differences especially in CT. Recent studies in Cognitive Neurodynamics field also discussed several variables that cater interest such as decision-making (18), and brain activity patterns and mental (19). Other than that, the gender differences also reported in spirituality well-being (20) and mental fatigue (21). All these factors open the door to relate the Cognitive Neurodynamics with CT for developing better students in local context.

Thus, the very design of the brain and the resulting disparities in sensory perception and physical skills differ considerably between the sexes. Understanding those variances will assist instructors in providing a good and encouraging environment for their pupils, as well as promoting CT through teaching and learning.

## Literature review

### Computational thinking

In today’s digital age, CT must be grasped quickly. CT is a kind of thinking that aligns with many 21st century abilities, such as problem-solving, creativity, and critical thinking (22). It derives from computer science and involves problem-solving, system design, and understanding people’s behavior (5). CT refers to the cognitive process of problem solving (23) as a set of 21st century skills (24, 25) or the thought process involved in formulating problems and expressing solutions (26) and a set of problem-solving skills based on Computer Science (27). This encouraged researchers to perform more in-depth research on learning experiences and computational thinking methods (28). Researchers couldn’t predict all difficulties before implementation (29).

CT is used from early childhood to university (30–32). The use of CT in formal education has taken numerous forms, including integration in computer science courses and embedding in math, science, and art (33, 34). (35) CT has also reached classrooms through robotics (36) and unplugged activities, such as board games or storybooks (37–39). While much has been said about demystifying CT pedagogy, research on evaluating CT skills and attitudes continues. To investigate systematic issues, it is indeed necessary to improve the attitude towards CT (40).

A review of the past five years (2016-2020) reveals that little research has been conducted on student CT disposition (41, 42). Correspondingly, attitudes affect CT as much as skills (5). CT’s complexity inspires others to investigate further, implying a deeper understanding of CT as a disposition (43, 44). In the digital age, self-directed problem-solving instruction may no longer be adequate. This problem-solving method does not account for the willingness to incorporate these abilities. Thus, researchers propose that CT dispositions are crucial motivators for identifying complex real-world issues and developing effective solutions (45, 46). According to the National Research Council (NRC), specific thinking skills are associated with an innate desire to think and are constituents of specific thinking dispositions (47). Thus, good thinkers possess the ability to think and the disposition to think (48). It appears that a validated evaluation of CT dispositions is lacking.

Notwithstanding, there is a persistent desire in all fields to distinguish between various conceptualizations of CT measurement. Psychometric scales are one of the most often employed instruments for evaluating computational thinking (49–53). However, psychometric instruments are predicated on the notion that an individual provides accurate and comprehensive information (54). On the other hand, a western instrument might not be suitable for Malaysia, given the distinct cultural and geographic environments. As a result, an instrument that has already been verified might not be reliable in a different period, culture, or setting (55–57). Additionally, it can be difficult to compare data from various cultures and groups when studying attitudes (58).

Furthermore, it is a well-known fact that the issue of gender inequality in CT is coming up more frequently. Every student exhibits a different level of CT proficiency depending on their location, gender, and academic standing, as is well known (59). The CT of males and females is essentially the same. Although earlier studies (60–62) found no differences in CT skills between male and female students aged 15 to 18, gender inequalities still exist (6, 60). CT skillset is frequently correlated with mathematical reasoning, favoring male students (59). Researchers explained the contradictory results, suggesting that the task content might be to blame for the differences. For some tasks, boys or girls may find them more interesting (63). This implies that earlier research on gender issues has produced conflicting findings, demonstrating the need for additional study.

In Cognitive Neurodynamics aspect of human development, one of the important aspects for behavioral, cognitive, and neural sciences is related to decision-making (18). The complexity of real-life decision-making has the potential to be linked to one’s person CT abilities. When a person is able to master CT well, then there is a possibility that their decision-making abilities also increase. CT may relate to brain activity patterns and mental. This point of discussion supports the findings of previous studies that there is a systematic link between brain activity patterns and spontaneously generated internal mental states (19).

In the context of this study, a person’s gender in CT also encourages in effecting spirituality well-being. (20) in his study found the existence of a gender effect on spirituality and showed that alpha and theta brain signals increased in male students at the 30–35 age range; while this increase was slower at the 20-29 age range. External factors such as decision-making, brain activity patterns, internal mental, and spirituality also can be linked to a person’s gender in CT differences. In addition, a study by Sadeghian et al. (21) also discusses mental fatigue based on gender. Their findings strengthen previous studies by showing the existence of a significant difference between the two groups of men and women for brain indicators with the alpha-1 index in men was higher than women and the average alpha-2 index in women was higher (both alpha indexes were to measure mental fatigue). This means that this difference in mental fatigue also has the potential to be linked to a person’s CT ability according to gender.

However, most CT measurement methods focus on thinking skills rather than dispositions. The architecture of the CT disposition measurement model suggested in this paper is built on cognitive, affective, and conative. In this perspective, the study’s importance can be viewed differently. It’s important for developing a measurement tool’s item pool and similar questions. It also helps create content for the most common components in modern literature. In many studies, limited tools such as perception-attitude scales, multiple choice tests, or just coding have been used to measure computational thinking (49, 51, 52, 64–66). This study established the Computational Thinking Disposition Instrument (CTDI) by considering several aspects of computational thinking.

### Computational thinking disposition

The development of CT dispositions necessitates long-term involvement in computational techniques focused on the CT process (67). CT’s psychological makeup remains a mystery to this day (52). When it comes to the internal impulse to act toward CT or respond in habitual but adaptive ways to people, events, or circumstances, the disposition is the term that describes it (68). While CT is most often regarded as a problem-solving process that emphasizes one’s cognitive process and thinking skills (69, 70), more attention should be paid to the dispositions that students develop in CT education. CT dispositions refer to people’s psychological status or attitudes when they are engaged in CT development (71). CT dispositions have recently been referred to as “confidence in dealing with complexity, a persistent working with difficulties, an ability to handle open-ended problems” (33, 72). Social psychologists describe dispositional traits as having an “attitudinal tendency” (73–75). Thoughtful dispositions, on the other hand, are often described in the context of critical thinking as a “mental frame or habit” (76). Furthermore, theorists argue that thinking is a collection of dispositions rather than knowledge or skill and that this is the case (77, 78).

Three psychological components comprise disposition: cognitive, affective, and conative. These three components of the mind are traditionally identified and studied by psychology (79–81). Information is encoded, perceived, stored, processed, and retrieved during cognition. A dispositional cognitive function is an individual’s propensity to engage in cognitive mental activities such as perception, recognition, conception, judgment, and others. Affection is the emotional interpretation of sensations, data, or knowledge. People, things, and concepts are frequently associated with one’s positive or negative relationships, and the question “How do I feel about this information or knowledge?” Self-actualization/self-satisfaction determines whether or not students feel successful after practicing CT in problem-solving exercises.

In contrast, conation refers to the relationship between knowledge, emotion, and behavior, which is ideally positive (rather than reactive or habitual) behavior (82, 83). Conative mental functions are “that aspect of mental activity that tends to develop into something else, such as the desire to act or a deliberate effort.” Determination to an endeavor is a conative mental capacity. In this investigation, these attitudes and dispositions serve as theoretical entities. Different contexts and requirements necessitate distinct mental dispositions, according to the study’s findings.

Due to the paucity of research and development on this topic, this study will make a substantial contribution to the body of knowledge as a result of its focus on computational thinking. Additionally, the tool gave an alternate perspective for evaluating students’ success in the CT course. In response, we aim to take a psychometric approach to these challenges. On the other hand, the creation and development of our Computational Thinking Disposition Instrument is described, along with its descriptive statistics and dependability based on its administration to more than 500 Malaysian students. Consequently, the purpose of this work is to provide a novel instrument for assessing CT and to demonstrate the relationships between CT and other well-established psychological dimensions.

### Research question

The purpose of this paper is to answer the following research question, which focuses on gender variations in attitudes concerning CT. Following the discussion on computational thinking disposition, a research question guides this paper:

1. To identify the existence of GDIF items in the Computational Thinking Disposition Instrument.

## Methodology

### Sample

The study employed a quantitative cross-sectional survey to collect and numerically analyze data to better comprehend the events under investigation (84). A self-administered online survey was used to collect the data, saving money, time, and effort. So, the data are almost ready for statistical analysis (85). The questionnaire survey was utilized since it is acceptable for a high sample size with a broad geographical coverage (86). This method also required respondents to check all boxes before submitting their responses, thereby minimizing data gaps. This study was conducted with the participation of 535 secondary school students with a background in computer science. Using probability sampling, samples were generated. Probability sampling employs a method of random selection that permits the estimation of sampling error, hence decreasing selection bias. Using a random sample, it is possible to describe quantitatively the relationship between the sample and the underlying population, giving the range of values, called confidence intervals, in which the true population parameter is likely to lie (87). Respondents were required to have a background in computer science, be willing to fill out questionnaires, and engage in online activities. The research was performed in October of 2020. Regarding ethical considerations, the student’s permission to participate in this study was obtained prior to completing the questionnaire. Participation was voluntary and strictly anonymous. Table 1 displays the demographic profile of the respondents.

**TABLE 1:** Demographic profile.

Demographic factor	Frequency	Percentage (%)	Total
**Gender**			
Male	247	46	535
Female	288	54	

### Instrumentation

A Computational Thinking Disposition Instrument (CTDI) measures students’ disposition in computational thinking. As was previously noted, three components were used to design the CTDI questionnaire. Sovey et al. (88) used factor analysis to demonstrate the items and validity constructions for the three constructs. EFA was the starting point of the investigation, then Rasch. The CTDI includes three demographic questions (gender, location, and prior knowledge) and 55 items in three dimensions that measure computational thinking disposition such as Cognitive (19 items), affective (17 items), and conative (19 items). All items had a 4-point Likert scale from strongly disagree (1) to strongly agree (4). Hence, there are recommendations that odd-numbered response scales should be avoided (89, 90). Dolnicar et al. (91) have explained that odd five-point Likert scales affect response styles that are biased, lack stability and take a long time to complete. The middle point scale category encourages a disproportionate number of responses (because the tendency to choose the middle scale is high). In the context of the study, the firmness of the respondent is considered an important basis in answering the items. Therefore, Sumintono and Widhiarso (92) have suggested not to provide a midpoint option. This argument is also supported by Wang et al. (93) who recommend that the midpoint scale not be used to obtain the views of Asian respondents. The scale is more appropriate compared to the conventional scoring method for the use of the Rasch model in this study. Ten pupils in total were then chosen for face validation. They were tasked with locating and cataloguing any unclear word or terminology. Additionally, they were permitted to share their thoughts on how to improve the questionnaires’ quality in terms of font size and design so that the research sample could understand them more quickly. These 10 pupils were left out of the main study.

On the other hand, Rasch measurement model software WINSTEPS 3.73 was used to determine the instrument’s validity and reliability. Rasch analysis (94) uses assumptions and a functional form to determine if a single latent trait drives questionnaire item responses. The Rasch model shows the assumed probability of participants’ scale response patterns, which are added and tested against a probabilistic model (94, 95). The Rasch rating scale analysis model is used when a set of items share a fixed response rating scale format (e.g., Likert scale) and thresholds do not vary. Through its calibration of item difficulties and person abilities, the WINSTEPS software transformed raw ordinal data (Likert-type data), based on the frequency of response which appeared as probability, to logit (log odd unit) via the logarithm function, which assesses the overall fit of the instrument as well as person fit (96, 97). Rasch models are used in this study to determine gender. Bond and Fox (98, 99) propose three DIF indicators for groups that have been studied: (1) *t* value ± 2.0 (−2.0 ≤ *t* ≤ 2.0), (2) DIF Contrast ± 0.5 (−0.5 ≤ DIF Contrast ≤ 0.5), and (3) *p* < 0.05.

### Person reliability and item reliability

According to Table 2, the “real” Person Reliability index (above 0.8) demonstrates that the consistency of individual responses was satisfactory (97). This indicates that the scale discriminates between individuals very well. This indicates that the likelihood of individuals responding to items was likely high. The same interpretation logic applies to Item Reliability measurements exceeding 0.90, which are also categorized as “very good” (100). High estimates of item reliability also indicate that the items define the latent variable very well (97). The CTDI may be considered a reliable instrument for various respondent groups.

**TABLE 2:** Reliability index and separation index.

	Respondent		Item		Cronbach Alpha
	Reliability index	Separation index	Reliability index	Separation index	
Cognitive	0.88	2.67	0.97	5.65	0.92
Affective	0.87	2.57	0.98	6.86	0.93
Conative	0.88	2.77	0.99	8.70	0.94

### Cronbach Alpha

The Cronbach Alpha coefficient value, as calculated by the Rasch Model, described the interaction between the 535 participants and the 55 items. According to Sumintono and Widhiarso’s instrument quality criteria, a reliability score of more than 0.90 (Table 3) is considered “very good” (2014). This result indicates a high degree of interaction between the people and the items. An instrument is highly reliable if it has good psychometric internal consistency.

**TABLE 3:** Analysis of GDIF items.

Construct	Number of items	Items exhibit GDIF contrast	Direction of GDIF	
			Male	Female
Cognitive	19	1	-	1
Affective	17	1	-	1
Conative	19	7	4	3

### Person and item separation index

The person Separation index measures how well the CTDI can distinguish between ‘Person abilities.’ Item Separation index shows easy and difficult items’ commonness (101). Wider is better. Bond and Fox (97) report that the item separation index is between 5.0 and 8.0, exceeding 2.0. Statistically, CTDI items could be divided into 5 to 8 endorsement levels. For respondents, a separation index above 2.0 is acceptable (102). Table 3 shows each construct’s internal reliability. These criteria endorse the CTDI as a reliable instrument for assessing students’ computational thinking disposition.

## Results

### Students’ disposition towards computational thinking

Firstly, students’ disposition towards computational thinking was analyzed. According to Table 4, among the three dimensions of disposition for computational thinking, students rated highest on affective, with a mean score of M = 2.76, SD = 2.08. However, lowest on the cognitive aspect, with a mean score of M = 2.48 and SD = 1.80. The results are summarized in Table 5.

**TABLE 4:** GDIF analysis of cognitive construct.

Construct	Item	*t*-value	Probability (Welch)	GDIF size	The direction of item GDIF
Cognitive	K3	0.00	1.0000	0.00	Free
	K5	−0.89	0.3756	−0.15	Free
	K6	0.88	0.3795	0.15	Free
	K10	0.30	0.7679	0.05	Free
	K21	−0.14	0.8872	−0.02	Free
	K26	−1.11	0.2678	−0.20	Free
	K29	0.29	0.7717	0.05	Free
	K31	1.46	0.1460	0.26	Free
	K32	0.00	1.0000	0.00	Free
	K33	−0.47	0.6409	−0.09	Free
	K34	0.72	0.4748	0.12	Free
	K35	−1.35	0.1777	−0.23	Free
	K37	−1.23	0.2189	−0.21	Free
	K38	−0.96	0.3397	−0.16	Free
	K43	1.78	0.0760	0.30	Free
	K46	−0.79	0.4326	−0.13	Free
	K49	0.72	0.4724	0.12	Free
	K50	−1.29	0.1989	−0.22	Free
	K52	2.19	0.0287	0.38	Female

The colored cells mean that the items colored were biased to gender. The values are not fulfilled the t-value (±2.00) and the probability (Welch) (>0.05).

**TABLE 5:** Descriptive statistics.

	Mean	Std. deviation
Cognitive	3.2410	0.4168
Affective	3.2903	0.4556
Conative	3.2771	0.4653

### Differences between students’ demographic factors and computational thinking disposition

GDIF analysis is performed to determine biased items in the CTDI instrument. Table 3 shows the summary of GDIF items in each construct of CTDI. With the critical *t*-value set at 2.0 and the confidence level at 95%, nine items were identified as significant for GDIF, extending the analysis to identify the extreme level of GDIF that could exist in the items. Using the Rasch Model, we can predict which items are likely to exhibit biases and eliminate the most significant DIF-exhibiting items to improve test fairness. The negative t-value and GDIF size indicate that male students answered the questions more easily than female students. Four (44.4%) of the nine items indicating the existence of GDIF were easier for males, while five (55.6%) were easier for females. There is a sizeable proportion of items that appeal to both genders. The disparities between the sexes are minimal, and the business’s direction is nonsystematic across all constructs. When the bias direction is not systematic, the moderately biased items are not problematic. The study revealed that item bias does not diminish the overall measurement accuracy and predictive validity of a test (103). As there is no benefit to removing these items, there should be a relatively high proportion of items in both ability groups that exhibit moderate DIF and have a low tendency to affect the instrument’s quality.

**FIGURE 1:**
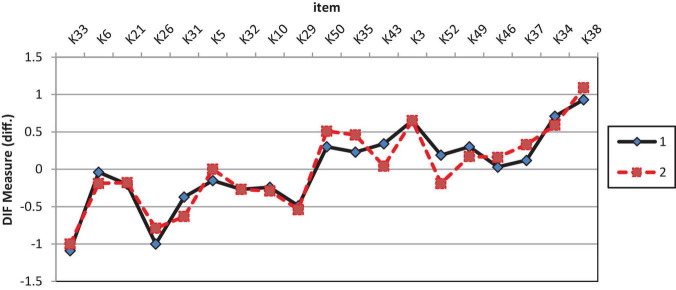
GDIF plot of cognitive items.

Table 4 shows the results of GDIF analysis on cognitive items. Analysis revealed that only one of 19 items showed significant GDIF, K52. Item K52 (“I know the importance of citing reference sources for assignments undertaken”) is easier to agree with by female students than male students. This item with a significant GDIF of 0.38 logits has a *t*-value of more than 2 (*t* ≥ 2.0). Figure 1 shows the DIF plot using the DIF measure on the analysis of cognitive construct by gender where 1 indicate male students and 2 refers female students.

**FIGURE 2:**
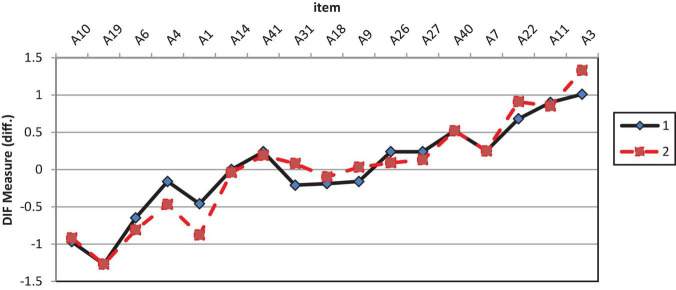
GDIF plot of affective items.

Table 6 shows the results of GDIF analysis on affective items. Analysis revealed that only one of 17 items showed significant GDIF, A1. Item A1 (“I do have a curiosity to explore new knowledge”) is easier to agree with by female students than male students. This item with a significant GDIF of 0.42 logits has a *t*-value of more than 2 (*t* ≥ 2.0). Figure 2 shows the DIF plot analysis of affective construct by gender (1 Male; 2 Female).

**TABLE 6:** GDIF analysis of affective construct.

Construct	Item	*t*-value	Probability (Welch)	GDIF size	The direction of item GDIF
Affective	A1	2.24	0.0256	0.42	Female
	A3	−1.95	0.0517	−0.33	Free
	A4	1.74	0.0833	0.32	Free
	A6	0.89	0.3751	0.17	Free
	A7	0.00	1.000	0.00	Free
	A9	−1.04	0.3001	−0.19	Free
	A10	−0.30	0.7617	−0.06	Free
	A11	0.31	0.7605	0.05	Free
	A14	0.25	0.8026	0.04	Free
	A18	0.13	0.8986	0.02	Free
	A19	0.00	1.000	0.00	Free
	A22	−1.37	0.1723	−0.23	Free
	A26	0.85	0.3964	0.15	Free
	A27	0.60	0.5475	0.11	Free
	A31	−0.09	0.6270	−0.28	Free
	A40	0.00	1.000	0.00	Free
	A41	0.27	0.7842	0.05	Free

The colored cells mean that the items colored were biased to gender. The values are not fulfilled the t-value (±2.00) and the probability (Welch) (>0.05).

**FIGURE 3:**
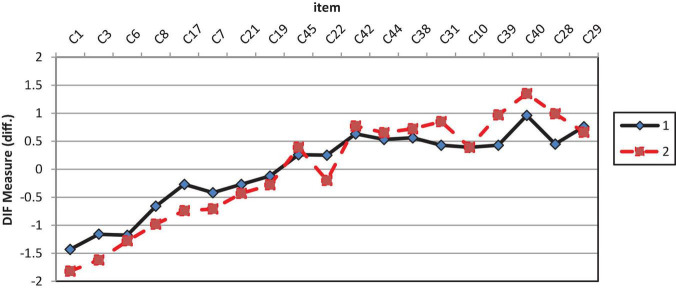
GDIF plot of conative items.

Table 7 shows the results of GDIF analysis on conative items. Analysis revealed that seven of 19 items showed significant GDIF, which are C3, C17, C22, C28, C31, C39, and C40. These items with significant GDIF ranging from 0.45 to 0.54 logits have a *t*-value of more than 2 (*t* ≥ 2.0). Figure 3 shows the DIF plot DIF measure analysis of conative construct by gender. Item C3 (“I am willing to tolerate current group members during problem solving”) is easier to agree with by female students than male students. Similarly, Item C17 (“I try to find the cause when a solution doesn’t work”) is easier to agree with by female students compared to male students. Additionally, Item C22 (“I diligently deal with a problem even beyond the allotted time”) is easier to agree with by female students than male students. Conversely, Item C28 (“I am willing to take risks to solve a problem”) is easier to agree with my male students than with female students. In addition, Item C31 (“I can adapt to the uncertainty of solving a problem”) is easier to agree with male students than female students. Item C39 (“I have the courage to accept challenges to solve complex problems”) is easier to agree with male students than female students. Item C40 (“I am confident that I understand the content of Computational Thinking”) male students were more confident in understanding the content of computational thinking.

**TABLE 7:** GDIF analysis of conative construct.

Construct	Item	*t*-value	Probability (Welch)	GDIF size	The direction of item GDIF
Conative	C1	1.95	0.0522	0.40	Free
	C3	2.32	0.0205	0.46	Female
	C6	0.52	0.6037	0.10	Free
	C7	1.56	0.1200	0.29	Free
	C8	1.70	0.0907	0.32	Free
	C10	0.00	1.0000	0.00	Free
	C17	2.52	0.0122	0.47	Female
	C19	0.89	0.3734	0.16	Free
	C21	0.87	0.3867	0.16	Free
	C22	2.49	0.0130	0.45	Female
	C28	−3.12	0.0019	−0.54	Male
	C29	0.54	0.5899	0.09	Free
	C31	−2.44	0.0150	−0.42	Male
	C38	−0.90	0.3680	−0.16	Free
	C39	−3.14	0.0018	−0.54	Male
	C40	−2.27	0.0236	−0.38	Male
	C42	−0.84	0.4020	−0.14	Free
	C44	−0.70	0.4870	−0.12	Free
	C45	−0.70	0.4836	−0.12	Free

The colored cells mean that the items colored were biased to gender. The values are not fulfilled the t-value (±2.00) and the probability (Welch) (>0.05).

## Discussion

This advancement in science and technology has not had the same effect on men as it has on women. Differential item functioning (DIF) is present when two or more subgroups perform differently on a test item despite being matched on a measured construct. DIF analysis plays a crucial role in ensuring the equity and fairness of educational assessments since DIF-free instruments are regarded as equitable and fair for all participants. Consequently, the DIF study is a crucial procedure that aims to identify the item that does not demonstrate the same function when administered to students with the same ability but different backgrounds.

Nine out of the 55 items associated to gender in total do not fall within the acceptable range, hence it is suggested that they can be removed (99). The value was between 0.42 and 0.46, according to the DIF contrast results in Tables 4, 6, 7, while the t value was between 1.95 and 1.95 logits. The result is in agreement with the logit value of +0.5 to 0.5 determined for the DIF contrast for the Likert scale and the t value between 2 and +2. (97, 104). Apart from that, since the probability was higher than 0.05, these items did not include DIF (92). In general, geographic location, gender, and academic achievement affect a student’s skillset (59). Gender differences are also a notable discussion in CT study (105). However, certain studies also indicated that males and females have similar CT. Despite no difference in CT between male and female 15-18-year-olds (61, 62), gender inequalities persist (6, 60).

Regarding the cognitive construct, K43 (I can change my mind to try something new while solving a problem) demonstrates that male students have superior cognitive abilities compared to female students. Boys are stronger at deductive and abstract reasoning, whereas girls are better at inductive and concrete (15). Boys reason from the general to the specific. They employ concepts to solve problems. Male brains are 10-15% larger and heavier than female brains, according to study. Besides size, genders also differ in brain autonomy. Using brain mapping, researchers found that men have six times more gray matter connected to intelligence than women, but women have ten times more white matter. One study shows that gender-related differences in brain areas connect with IQ (106). This study and others show that males’ inferior parietal lobes are larger. This lobe helps boys with spatial and mathematical reasoning. The left side of the brain, which controls language and verbal and written skills, develops sooner in girls, so they perform better in those areas (107). These results concur with Mouza et al. (108) ‘s conclusion that male students have a higher cognitive level of CT knowledge than female students. In addition, other studies have found that female students have limited computing knowledge and experience (109). According to research, males are typically more interested in information or knowledge than females (110). This may be due to the influence of culture and stereotypical socialization processes experienced by people beginning in childhood, as there are more men than women in these sectors (61). Lack of early experience and other obstacles contribute to girls’ underrepresentation in this field (111).

Examining the affective construct, findings indicate that male students are more interested in practicing CT than female students for items A4 (I want to learn programming to apply Computational Thinking) and A1 (I have a curiosity to explore new knowledge). Similarly, Askar and Davenport (112) and Ozyurt and Ozyurt (113) found that male students have a greater sense of programming self-efficacy than female students. In addition, other studies have shown that the lack of female role models and differences in prior programming experience influence women’s participation in computer science (114, 115). In addition, CT aptitude appears to be frequently linked to mathematical logic and favors male students (59). In addition, a study conducted outside of Malaysia revealed that male students were more familiar with technology and favored its use for learning (116). Female students typically require more time than male students to master CT (60). Atmatzidou and Demetriadis (60) reported that girls in the high school robotics STEM curriculum appeared to require more training time to attain the same skill level as boys in certain CT-specific aspects, such as decomposition.

Moreover, in the conative construct, male students won item C17 (I try to find the cause when a solution does not work). According to neuroscience study (117–119), females’ hippocampus develops faster and is larger than boys. Sequencing, vocabulary, reading, and writing are affected. Boys learn better through movement and visual experience because their cerebral cortex is more defined for spatial relationships. Girls favor collaborative activities where they exchange ideas with others, while guys prefer rapid, individualistic, kinesthetic, spatially-oriented, and manipulative-based activities (120). This fits with what Geary et al. (121) found: that male and female students have very different spatial and computational skills because male students are better at arithmetic reasoning. It is influenced by the fact that male students tend to be more intellectual, abstract, and objective. As a result, male students tend to understand issues through calculations, evaluate the compatibility between computational tools and techniques and challenges, and use computational strategies when solving problems. Besides, the additional information in Cognitive Neurodynamics context showed the existence of a gender effect on spirituality which reported that alpha and theta brain signals started increased in male according by age range (20). Computational thinking is breaking down complicated problems into steps that can be understood (called algorithms) and finding patterns that can be used to solve other problems (22). On the other hand, female students worked harder to find the first information. Still, female students tended to solve problems step by step, making it hard for them to find patterns or quick ways to solve problems. Women tend to be more careful, organized, and thorough than men (122, 123). Overall, the existing results proved that the existence of a significant difference between the two groups of men and women for brain indicators in the Cognitive Neurodynamics context.

## Limitations and future directions

This study, like any other, has limitations. To begin with, this research is only focused on computational thinking disposition aspect. As a result, the CTDI instrument was built around three main elements in disposition. Thus, the first limitation is only Malaysian secondary school students were included in the study. Context can affect cultural differences. Researchers assert that context may explain the different results. Therefore, it is best to conduct larger-scale research with samples across Malaysia. This would increase the respondents’ and research’s demographic diversity.

DIF benchmarking could include in secondary schools. More research is needed to understand the differences in DIF item performance between groups, especially since computational thinking instruments are still being developed. We based our work on CT literature from various domains. The tool should apply to other fields. Replications in other countries would boost relevance in diverse nations. This study’s construct validity came from a homogenous population. The scale must be validated with higher education, elementary, middle, and private school students. Comparing studies across tests can also improve psychometric assessment.

On the other hand, there are multiple areas for further research that stem from this study. Accordingly, future research should include different cultural groups to determine if the phenomenon is universal. East Asians and Westerners have different thinking patterns, according to Nisbett et al. (124). Westerners strongly prefer positivity, while Easterners have more varied preferences (125). This study will influence future analyses and improve item psychometrics. Further research can correlate personality traits. This instrument is DIF-analyzed in psychometrics to ensure it has not biased towards one measurement component as ethnic, socioeconomic status or age groups may contribute to the DIF.

## Conclusion

This study assessed the effects of gender differences on disposition towards CT Using DIF analysis. Implementing a curriculum design to integrate STEM education with computational thinking to create an interdisciplinary approach presented a number of obstacles. A well-organized measuring instrument should be designed for long-term utilization. The information on gender clustering tendencies in answering GDIF items can help test developers create more fair achievement test items for students of different genders. The important practical implication is that the items selected from this study can be used as an alternative for self-evaluation and peer evaluation session for improvement purposes.

The findings of this study revealed a moderate level of CT disposition, which suggests the importance of making students aware of the evolution and rapid growth of CT discipline, and the availability of technological resources. The DIF analysis showed that there was a significant difference based on gender towards students’ disposition for CT. Educators can use the data to identify students’ strengths and weaknesses and plan more meaningful lessons. Girls and boys alike can flourish in their creative thinking if we teach them to focus on the process of CT and problem solving.

We need to acknowledge the fact that boys’ and girls’ perspectives on CT differ in significant ways. Differences in ability are not included in these categories. In order to encourage excellence in both sexes, educators must take into account the differences between males and females while planning lessons and activities. The necessity of devising engaging interventions and monitoring children’s attentional and motivational elements during activities is illuminated by these findings, which have implications for educational practitioners and researchers. The study makes CT dispositions visible to the education community as path-opening invitations to explore CT and foster meaningful learning experiences.

## Data availability statement

The original contributions presented in this study are included in the article/supplementary material, further inquiries can be directed to the corresponding author.

## Ethics statement

The studies involving human participants were reviewed and approved by Education Policy Planning and Research Division, Ministry of Education, Malaysia (Ethics approval number: KPM.600-3/2/3-eras (7906). Written informed consent to participate in this study was provided by the participants’ legal guardian/next of kin.

## Author contributions

SS: conceptualization, methodology, formal analysis, investigation, writing—original draft preparation, and project administration. SS and MM: validation and data curation. SS, MM, and KO: resources and writing—review and editing. MM: visualization and funding acquisition. MM and KO: supervision. All authors read and agreed to the published version of the manuscript.
